# Adult Langerhans cell histiocytosis with pulmonary and colorectoanal involvement: a case report

**DOI:** 10.1186/s13256-017-1428-7

**Published:** 2017-09-25

**Authors:** Mohamad Jihad Mansour, Elias Mokbel, Eddy Fares, Janah Maddah, Fadi Nasr

**Affiliations:** 1 Lebanese University, Faculty of Medical Sciences, Hadath, Lebanon; 2 Mount Lebanon Hospital, Hazmieh, Lebanon

**Keywords:** Langerhans cell histiocytosis, Histiocytosis X, Multisystem involvement, Chemotherapy, Immunohistochemistry

## Abstract

**Background:**

Langerhans cell histiocytosis is a rare systemic disease characterized by the abnormal overproduction of histiocytes that tend to infiltrate single or multiple organ systems leading to significant tissue damage. It mainly affects – by order of decreasing frequency – the bone, the skin, the lymph nodes, the liver, and lungs. Gastrointestinal tract involvement is extremely rare in adults.

**Case presentation:**

We describe the case of a 32-year-old Middle Eastern man with Langerhans cell histiocytosis involving his lungs and the colorectoanal part of his gastrointestinal tract, with complete resolution of gastrointestinal tract lesions following a non-standardized chemotherapy regimen.

**Conclusions:**

Gastrointestinal tract lesions are a rare manifestation of Langerhans cell histiocytosis, especially when associated with extraintestinal involvement, such as the lungs. Chemotherapy protocols have not been well established for the treatment of the disease. The clinical impact of the effective chemotherapy regimen used to treat this uncommon presentation of Langerhans cell histiocytosis will be viewed in this case report.

**Electronic supplementary material:**

The online version of this article (doi:10.1186/s13256-017-1428-7) contains supplementary material, which is available to authorized users.

## Background

Langerhans cell histiocytosis (LCH) or histiocytosis X is a rare systemic disease characterized by the abnormal overproduction of histiocytes that tend to infiltrate tissues and organ systems leading to organ damage. Its estimated incidence in adults is approximately one to two cases per million persons [1] compared to three to five cases per million in the pediatric population. The pathogenesis of LCH is still under debate. It is unknown whether LCH is a reactive or neoplastic process. LCH is of reactive nature when remissions occur spontaneously [2]. On the other hand, the infiltration of organs by a monoclonal population of aberrant cells, the possibility of lethal evolution, and the cancer-based modalities of successful treatment are all consistent with a neoplastic process [2, 3]. Gastrointestinal (GI) tract involvement is extremely rare in adults.

In this case report, we highlight an unusual presentation of LCH and the effectiveness of a non-standardized chemotherapy protocol for the treatment of this rare disease. To the best of our knowledge, this is the first case report to describe pulmonary and GI tract involvement. Here we present the case of a 32-year-old man with LCH involving the lungs and the colorectoanal part of the GI tract, with complete resolution of GI tract lesions following chemotherapy.

## Case presentation

**Fig. 1:**
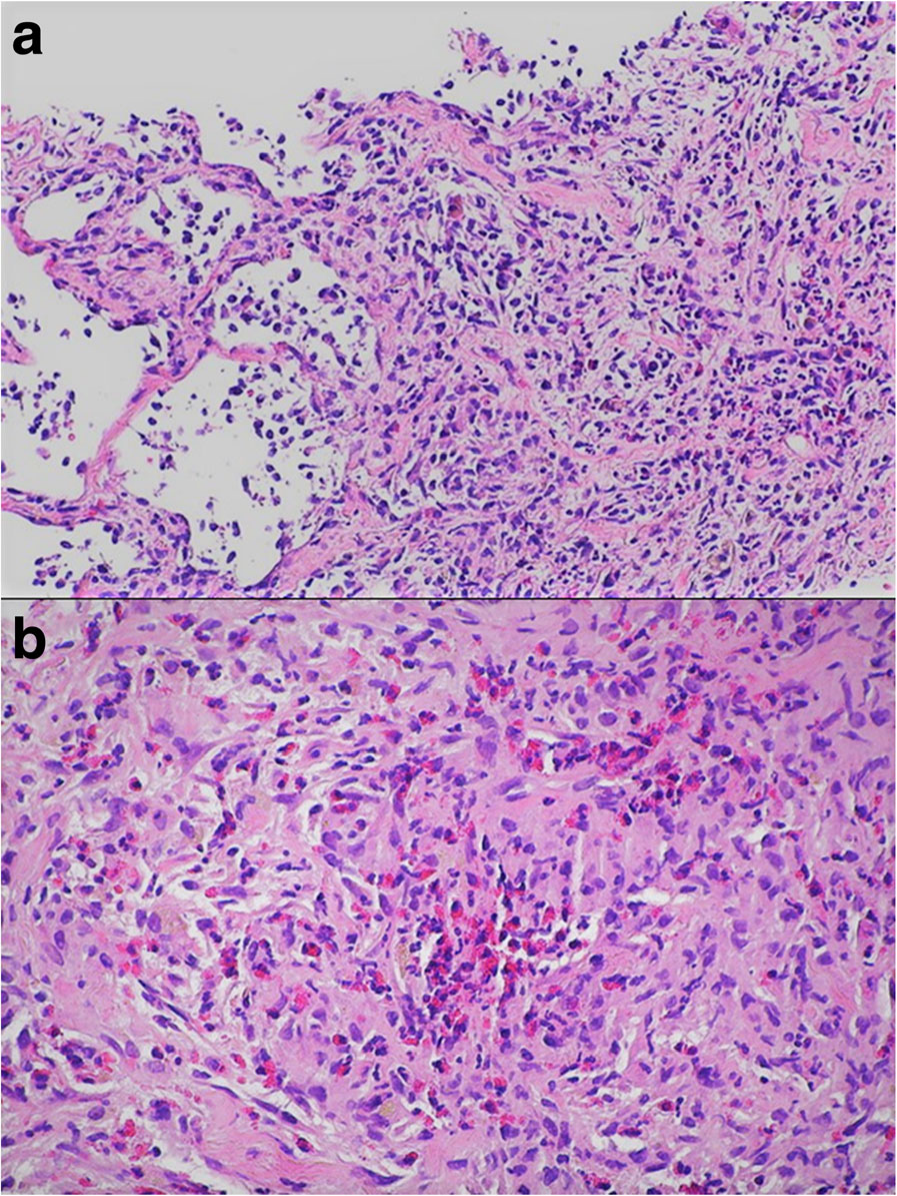
Pathology slides of a transbronchial biopsy specimen. **a** Intermediate magnification (× 100) showing proliferation of Langerhans cells in the pulmonary interstitium. **b** High power (× 400) transbronchial biopsy specimen showing a large infiltrate of Langerhans cells

A 32-year-old Middle Eastern man, who smoked 20 cigarettes per day and was an occasional alcohol consumer, with a family history of thyroid disease, presented to our emergency room with multiple painful anal lesions that started to appear a few weeks prior to presentation with occasional bleeding and purulent discharge. His past medical history was significant for diabetes insipidus 10 years ago after recurrent complaints of polyuria and polydipsia. At 26 years of age, he started complaining of non-productive cough and exertional dyspnea. A chest X-ray and computed tomography (CT) scan of his chest done at another institution showed the presence of bilateral pulmonary cystic lesions involving the upper lobes. He was misdiagnosed to have pulmonary fibrosis and chronic obstructive pulmonary disease and was started on high-dose orally administered prednisone therapy 2 years later, then received daily budesonide/formoterol without clinical improvement. Four years later, a high resolution multi-detector CT scan of his chest showed massive honeycombing cystic changes of both lung fields with intermingled fibrosis in between. Findings were suggestive of histiocytosis X. A transbronchial biopsy of the lung lesions confirmed the diagnosis of adult pulmonary LCH (Fig. 1).

**Fig. 2:**
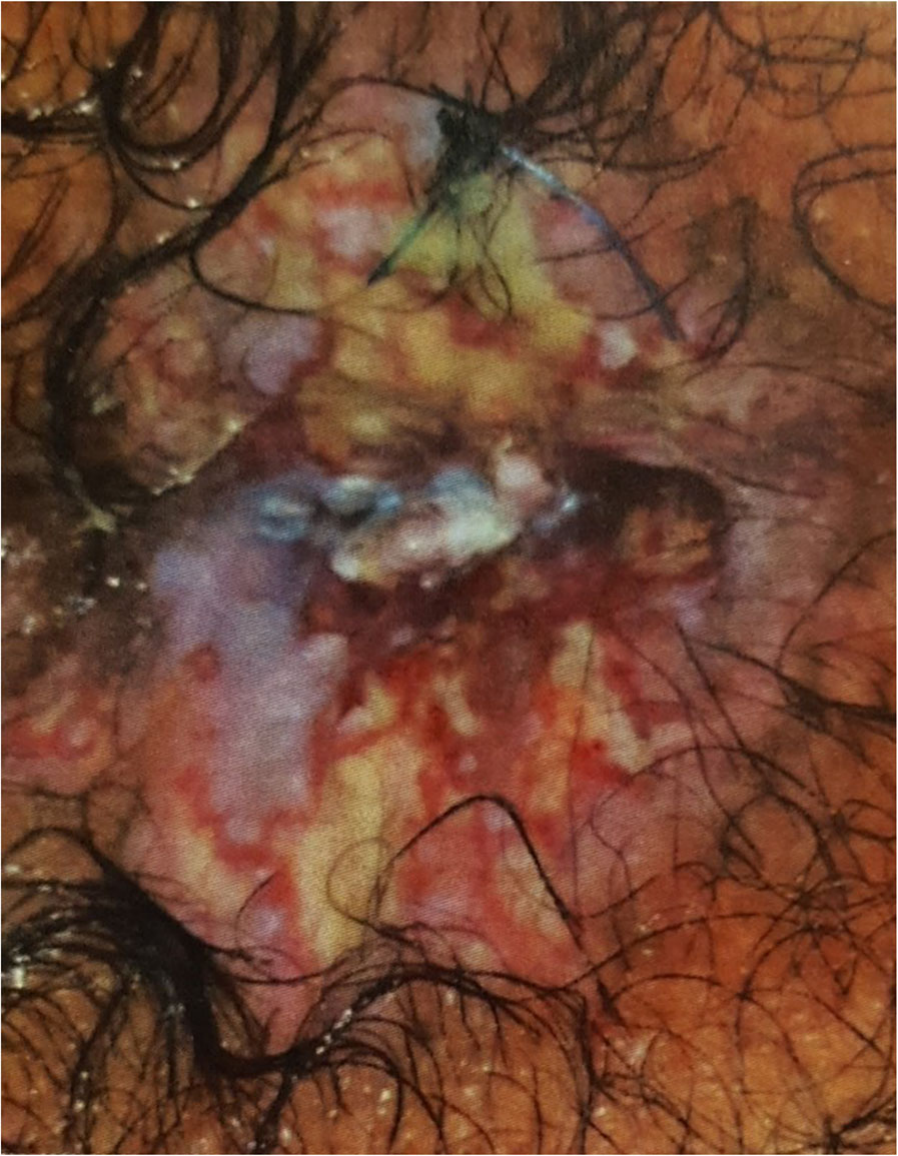
Perianal skin lesions due to Langerhans cell histiocytosis

On admission, physical examination revealed an ill-appearing man but he was not in acute distress. He was conscious and oriented. His vital signs were within normal limits except for tachycardia of 112 beats per minute. There were no palpable cervical or inguinal lymph nodes. Cardiac auscultation was remarkable for a rapid heart rate with regular S1 and S2 without murmur. Chest auscultation revealed bilateral scattered wheezes best heard at the upper lung fields. His abdomen was soft and non-tender. Macroscopic examination of his anal canal revealed a cutaneous lesion infiltrating the anal sphincter with circumferential perianal lesions and eroded ulcerative plaques over the anal orifice (Fig. 2).

He was admitted for further evaluation and management. A laboratory workup revealed a normal white cell count 7200/μL (normal range 4000 to 11,000/μL) with 76% neutrophils, normal levels of hemoglobin 16.1 g/dL, blood urea nitrogen 11 mg/dL, and creatinine 0.75 mg/dL. His C-reactive protein was 26 mg/L (normal < 10 mg/L). Liver function tests and electrolytes were all normal. Human immunodeficiency virus-1 (HIV-1) and human immunodeficiency virus-2 (HIV-2) antigens/antibodies (Ag/Ab), hepatitis C Ab, and hepatitis B surface Ag were within normal ranges (non-reactive 0.24, 0.08, and 0.23, respectively; negative if less than 1.0 and positive if more than 1.0). A urine analysis was negative for ketones, proteins, glucose, white cell and red cell counts, and bacteria. Throat, urine, stool, and rectal swab cultures were all negative.

Histopathologic examination and immunohistochemical staining of the anal lesion biopsy specimen showed the presence of histiocytoid cells that tested positive for CD1a and S100 protein, while CD3 and CD68 revealed scattered positivity, consistent with LCH. Periodic acid–Schiff (PAS), Gram, and acid-fast stain were non-revealing.

A colonoscopy showed the presence of an established circumferential, fungating rectal tumor of 1 cm diameter with no luminal obstruction.

**Fig. 3:**
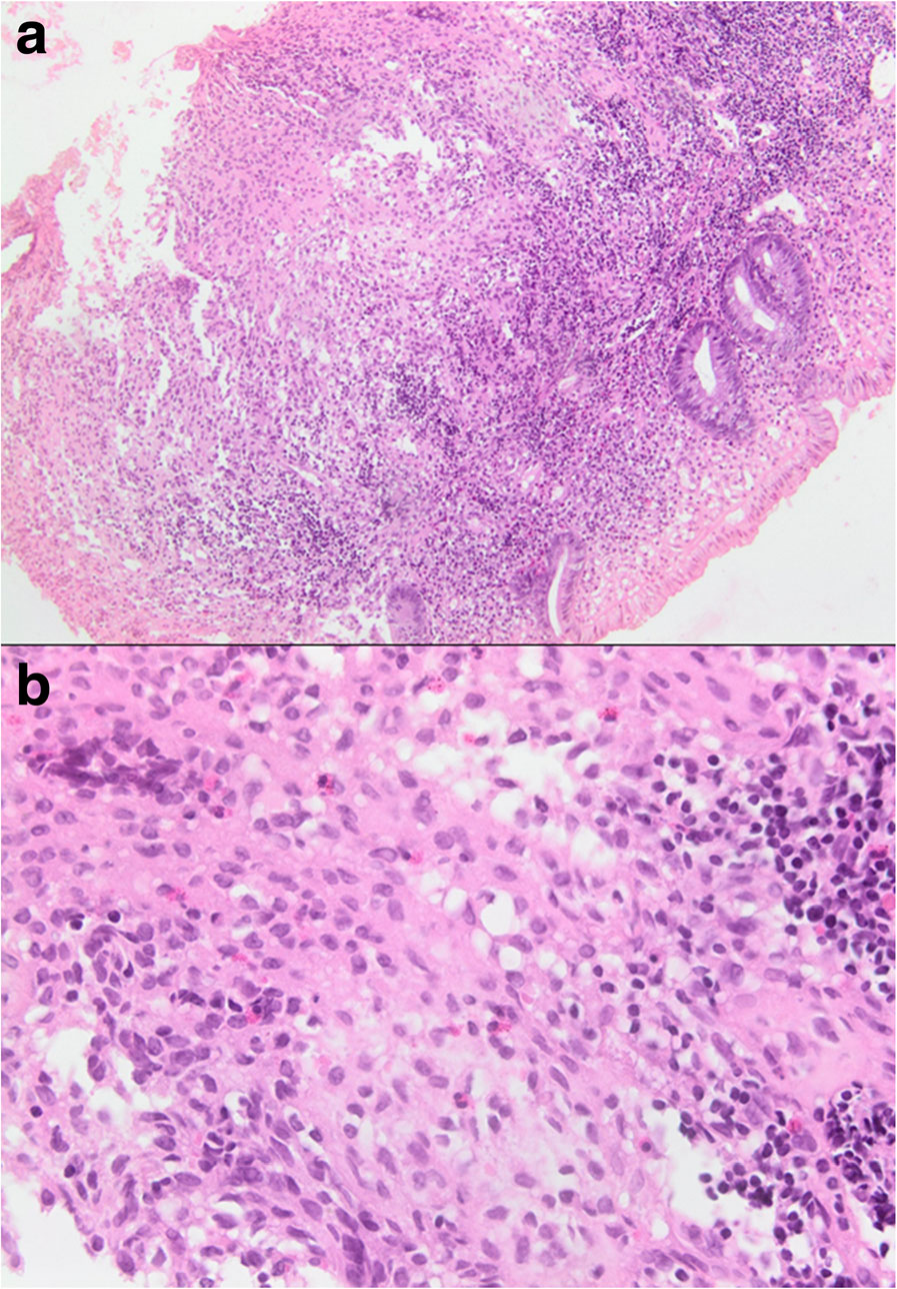
Biopsy from the sigmoid colon. **a** Colonic biopsy showing histiocytoid cells and abundant surrounding eosinophils and few lymphocytoid cells; × 100 magnification. **b** A colonic biopsy showing submucosal infiltration of Langerhans cells; × 400 magnification

**Fig. 4:**
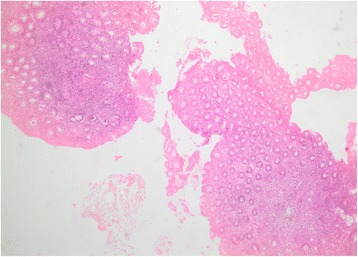
Microscopic examination of an anal polyp consistent with a hemorrhoidal fibroma

He received intravenously administered chemotherapy with vinblastine 10 mg on day 1 and day 15 along with orally administered prednisone 80 mg daily (1 mg/kg per day). After three cycles, magnetic resonance imaging of his abdomen and pelvis showed no evidence of disease in the rectosigmoid region. A CT scan of his chest revealed the presence of emphysema in both upper and lower lobes with fibrotic changes. He received two additional cycles of vinblastine 10 mg every 2 weeks then vinblastine 10 mg every 3 weeks. After four cycles of treatment, a CT scan of his chest, abdomen, and pelvis showed disease progression with honeycombing in both apices of his lungs with bilateral perianal infiltration. Colonoscopy revealed multiple colonic nodules from the rectum to the cecal region, with sigmoid ulceration. A histopathologic study of the sigmoid colon biopsy specimen showed a chorionic and submucosal infiltration with histiocytoid cells with clear cytoplasm and abundant surrounding eosinophils and few lymphocytoid cells consistent with the known diagnosis of histiocytosis X (Fig. 3). Histopathologic study of the anal biopsy specimen showed an acanthotic hyperparakeratotic mucosa that was discretely eroded with no evidence of malignant cells. He then received cytarabine 100 mg administered intravenously weekly plus vinblastine 10 mg administered intravenously every 2 weeks. After 14 cycles of treatment, re-evaluation with a CT scan of his chest, abdomen, and pelvis with intravenous contrast administration showed disease at the thoracic level with evidence of micronodules in the upper lobe of his right lung. At the pelvic level, there was no evidence of rectal tumor recurrence, with significant (>60%) decrease in the size of colonic nodules. Treatment was continued for an additional 15 cycles, with good treatment tolerance and no clinical evidence of vinblastine-related neurotoxicity. Orally administered steroids were slowly tapered to 10 mg daily. After the 15th cycle of treatment, he was hospitalized for rectorrhagia, but a CT scan of his chest, abdomen, and pelvis did not show any evidence of disease progression. On colonoscopy, a decrease in the size of the anal lesion was noticed without anal stenosis and the anal sphincter was less taken by the tumor. Repeat histopathologic examination of the colonic biopsy specimen showed persistent histiocytosis, but a biopsy of the anal polyp showed the presence of a hemorrhoidal fibroma (Fig. 4).

**Fig. 5:**
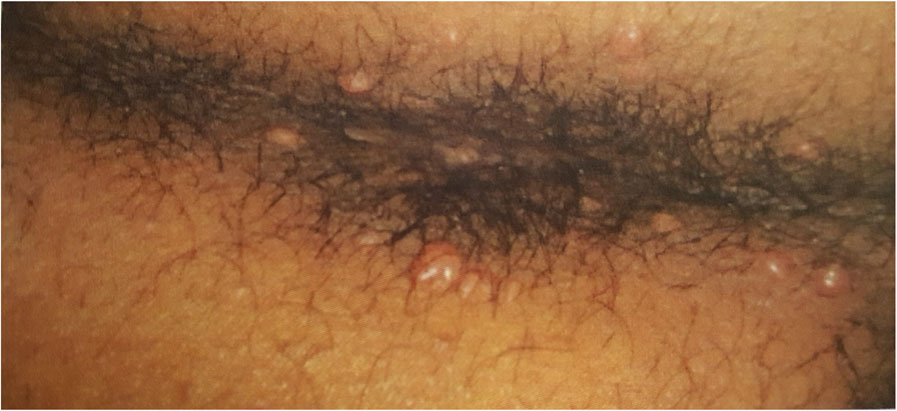
Anal orifice showing complete resolution of the histiocytic lesions with persistence of benign-appearing perianal micropolyps

After 3 weeks, he received gemcitabine (Gemzar) 1400 mg administered intravenously over 30 minutes and cisplatin 50 mg administered intravenously over 2 hours both every 14 days for eight cycles. The regimen was well tolerated with no hospitalizations for neutropenic fever or pancytopenia. A colonoscopy showed a normal colonic mucosa with disappearance of colorectal nodules, with few benign-appearing perianal micropolyps (Fig. 5). A repeat CT scan of his chest, abdomen, and pelvis with intravenously administered contrast confirmed the colonoscopic results with no evidence of anal and perianal lesions. Lung findings were stable compared to previous results. An additional file shows a timeline for our patient (see Additional file 1).

After completion of all cycles of chemotherapy, he was seen every 2 weeks in the out-patients clinic for a total of 6 months. He was clinically stable with no major complaints. Serial laboratory tests including a complete blood cell count with differentials, renal function, and electrolytes were within normal ranges. Follow up confirmed complete disappearance of the GI lesions and significant improvement of the pulmonary symptoms. Later, he was lost to follow up and could not be reached.

## Discussion

Adult LCH is rare and many cases remain undiagnosed. The age of onset in adults varied between 21 and 77 years [4, 5]. Some studies reported a female gender predominance [6], while others showed a slight male preponderance [4] or a more even gender distribution [7].

LCH can affect only one site in the body or may involve multiple sites or organs. One Germany-based registry reported LCH in adult patients affecting – in decreasing order of frequency – the bone, skin, pituitary, liver/spleen, brain, and the GI tract, the latter accounting for only 2% of cases [8].

Histiocytosis X originates from a myeloid dendritic cell giving rise to abnormal Langerhans cells that proliferate at a moderate rate. Histopathologic evaluation of suspicious lesions as well as immunohistochemical examination are necessary for a final diagnosis. Typically, histiocytic cells stain positive for CD1a and S100.

Clinical presentation depends on the site of involvement. In pulmonary LCH, patients often complain of non-productive cough and dyspnea, constitutional symptoms such as fever and weight loss, or eventually chest pain from spontaneous pneumothorax. Tobacco smoking was reported in many series to be a major contributory factor in pulmonary LCH [9–11]. In the GI tract, the stomach, small intestine, colon, rectum, and perianal skin may be affected [6, 12–14]. Most cases have been diagnosed by GI endoscopy, and up to 50% of patients were asymptomatic [6]. The distribution of the lesions in the GI tract varies, being most common in the colorectum [6]. In the majority of adults, LCH is typically encountered as a solitary polyp [6, 15], and usually occurs without multisystem involvement. However, isolated polyps in the stomach, small bowel, rectum, or colon are extremely rare, the latter being reported only in two cases [15] up to the year 2012. Multiple polyposis due to LCH of the stomach was reported in adults [15]. Lesions are predominantly intramucosal with either a marginated or infiltrative growth pattern. The overlying mucosa could become ulcerated or necrotic. The colonic submucosal infiltration seen in our patient is an unusual clinical finding. Anal involvement by LCH in the adult population is even rarer, and most reported cases were perianal lesions, around the anal orifice, and mainly limited to the perianal skin [12, 13]. Similar cases with anal fistula were reported in pediatric patients [14].

To the best of our knowledge, this is the first case of adult LCH involving the lungs and the colorectoanal part of the GI tract and presenting as multiple polyposis of the colon and rectum with multiple anal lesions infiltrating the anal sphincter and extending to the perianal region and skin, which showed complete response to chemotherapy.

The treatment of adult LCH is not standardized due to the rarity of the disease and the incomplete understanding of its pathophysiology. Vinblastine, a commonly used medication in pediatric LCH, is less favored in adults because of the risk of neurotoxicity. Despite this, our patient had a very good response to vinblastine but had disease recurrence once vinblastine administration was delayed to every 3 weeks instead of 2 weeks. Cytarabine at a dose of 100 mg/m^2^ for 5 consecutive days every month for a duration of 6 months has been used in adult LCH. Our patient received a total of 29 cycles of cytarabine plus vinblastine, with a very good clinical tolerability, without evidence of neurotoxicity or neutropenic fever, and had significantly less extensive involvement of the colorectum with partial regression of the anal lesion. After eight cycles of gemcitabine 1400 mg plus cisplatin 50 mg every 2 weeks, a chemotherapy regimen not previously used or reported for treatment of adult LCH, complete resolution of the colorectal, anal, and perianal lesions occurred. Treatment options in case reports concerning perianal involvement in LCH varied between surgical excision [13], successful use of thalidomide [16], Leustatin (cladribine), and radiotherapy with maintenance adiuretin [17], single agent imatinib or MACOP-B protocol (cyclophosphamide, doxorubicin, vincristine, methotrexate, bleomycin, and prednisone) with variable efficacy [18]. Combination gemcitabine plus cisplatin has not been previously reported and deserves further consideration in the management of similar lesions. Its use as first-line treatment in this group of patients has not been previously tested.

Overall prognosis in LCH seems to be favorable. A retrospective analysis [19] of 35 patients with LCH with multisystem involvement who received combination chemotherapy showed a 3-year overall survival of 81% ± 10% in patients aged 14 years and above. The overall survival of patients with LCH with GI tract involvement has not been specifically studied.

## Conclusions

Adult multiple polyposis in the colon, rectum, and anus due to LCH of the GI tract in association with pulmonary LCH is an extremely rare clinical entity. Chemotherapy remains the mainstay of treatment. Long-term cytarabine plus vinblastine has shown acceptable tolerability. Gemcitabine plus cisplatin combination chemotherapy is a reasonable treatment option that was associated with complete resolution of the GI tract lesions.

## Additional file

Additional file 1: Timeline. (DOCX 42 kb)

## Abbreviations

Ab: Antibodies
Ag: Antigens
CT: Computed tomography
GI: Gastrointestinal
LCH: Langerhans cell histiocytosis

## Authors’ information

FN is currently a lecturing ﻿P﻿rofessor at the Lebanese University, Faculty of Medical Sciences, Hadath, Lebanon, and the Head of Division of Hematology and Oncology at Mount Lebanon Hospital, Hazmieh, Lebanon.
