# Supplementary material for: Adult Langerhans cell histiocytosis with pulmonary and colorectoanal involvement: a case report
Source: J Med Case Rep. 2017 Sep 25;11:272. doi: 10.1186/s13256-017-1428-7 (PMC5611558; doi:10.1186/s13256-017-1428-7)
Supplement: Additional file 1: — Timeline. (DOCX 42 kb) [file 13256_2017_1428_MOESM1_ESM.docx]

Diabetes Insipidus (polyuria, polydipsia)

COPD? (non productive cough, exertional dyspnea, evaluated with a CT chest)

Lung fibrosis and honeycombing (CT chest). Histiocytosis X on transbronchial biopsy

10 years ago

Six years ago

Current illness

Multiple anal lesions (with bleeding and purulent discharge)

Anal biopsy and histopathologic examination: histiocytoid cells that tested positive for CD1a and S100 protein, while CD3 and CD68 revealed scattered positivity

- Histiocytosis X

Colonoscopy: rectal tumor of 1 cm diameter

Current illness

And management

chemotherapy with vinblastine day 1 of diagnosis and 15 days later + oral prednisone

2 additional cycles of vinblastine 10 mg every 2 weeks then vinblastine 10 mg every 3 weeks.

After 3 cycles of chemotherapy, MRI abdomen and pelvis: no rectosigmoid disease

CT scan of the chest revealed the presence of emphysema in both upper and lower lobes with fibrotic changes

CT scan of the chest, abdomen and pelvis: disease progression: honeycombing in both apices of the lungs with bilateral perianal infiltration.

Colonoscopy revealed multiple colonic nodules from the rectum to the cecal region, with sigmoid ulceration.

Histopathologic study of the sigmoid colon biopsy specimen showed a chorionic and submucosal infiltration with histiocytoid cells with clear cytoplasm and abundant surrounding eosinophils and few lymphocytoid cells consistent with the known diagnosis of histiocytosis X

Histopathologic study of the anal biopsy: acanthotic, hyperparakeratotic mucosa discretely eroded with no evidence of malignant cells

CT scan of the chest, abdomen and pelvis with IV contrast: disease at the thoracic level with evidence of micronodules in the right upper lobe. At the pelvic level, there was no evidence of rectal tumor recurrence, with significant decrease in the size of colonic nodules.

CT scan of the chest, abdomen and pelvis: no evidence of disease progression.

On colonoscopy: decrease in the size of the anal lesion without anal stenosis

cytarabine 100 mg intravenous (IV) weekly plus vinblastine 10 mg IV every 2 weeks for 14 cycles.

Treatment was continued for an additional 15 cycles.

After the 15^th^ cycle of treatment, admission for rectorrhagia

Repeat histopathologic examination of the colonic biopsy specimen showed persistent histiocytosis, but biopsy of the anal polyp showed the presence of a hemorrhoidal fibroma.

Colonoscopy showed a normal colonic mucosa with disappearance of colorectal nodules, with few benign appearing perianal micropolyps.

A repeat CT scan of the chest, abdomen and pelvis with IV contrast confirmed the colonoscopic results with no evidence of anal and perianal lesions. Lung findings were stable compared to previous results.

After three weeks, gemcitabine and cisplatine both every 14 days for 8 cycles.
